# Nutritional, Phytochemical, Mineral, and Morphological Variations in Hyacinth Bean (*Lablab purpureus* L.) Cultivars at Different Developmental Stages

**DOI:** 10.1002/fsn3.72086

**Published:** 2026-07-06

**Authors:** Gazi Wafa Akbar, Noshin Nower Bristy, Tania Ismail, Sabrina Mansur, Ahmed Saiyadeen Nusaha, Jobed Nahar, Rimon Barua, Afroze Anwar, Mubtasim Adnan, Nurul Absar

**Affiliations:** ^1^ Department of Biochemistry and Biotechnology University of Science and Technology Chittagong Chattogram Bangladesh

**Keywords:** hyacinth bean pods, *Lablab purpureus*, mineral composition, phytochemical analysis, pod developmental stage, South Asia

## Abstract

The hyacinth bean ( *Lablab purpureus* L.), is a major winter legume in Bangladesh. However, there is very little information on how different cultivars and developmental stages of this legume affect its nutritional profile concerning pod composition. Consequently, the purpose of this study was to determine how both genotype and developmental stage (maturity) affect the morphological characteristics, proximate composition, phytochemical characteristics, and mineral composition of hyacinth bean pods grown in Bangladesh. We examined six commercially grown genotypes, which included four improved BARI cultivars collected at three stages (immature, premature, and mature) from four different BARI research stations throughout Bangladesh, as well as two local varieties bought from Chattogram markets. The morphological characteristics were measured using precision measuring devices and included total stem and leaf area, total pod and seed weight, and pod length; proximate analysis included moisture content, crude protein, crude fat, crude fiber, and ash content; physicochemical analysis was accomplished using approved AOAC methods; for phytochemical analysis, we determined total phenolic content (TPC), total flavonoid content (TFC), and total Vitamin C content (TVC); finally, for mineral analysis we determined the amounts of calcium (Ca), potassium (K), phosphorus (P), and iron (Fe) in each genotype using atomic absorption spectrophotometry. In all cases, statistical analysis was conducted using two‐way analysis of variance (ANOVA) at *α* = 0.05. Significant genotype‐ and stage‐dependent differences were observed. Pod weight and dry matter increased with maturity, while moisture decreased. Potassium was the predominant mineral (780.3–2346.0 mg/kg DW). Immature pods exhibited relatively higher antioxidant‐associated phytochemicals. The study highlights the large amount of compositional diversity available among hyacinth bean cultivars and developmental periods, which indicates that this may have the potential to be a useful tool for crop selection and diet diversification in Bangladesh.

## Graphical abstract

For comparative analysis, six hyacinth bean cultivars (four BARI Varieties and two local cultivars) were evaluated into three different development stages that is, immature, premature and mature stage. The nutritional potential of both improved and traditional hyacinth bean cultivars in Bangladesh was highlighted by morphological, physicochemical, nutritional, phytochemical, and mineral analyses that exhibited significant cultivar‐ and maturity‐dependent differences.

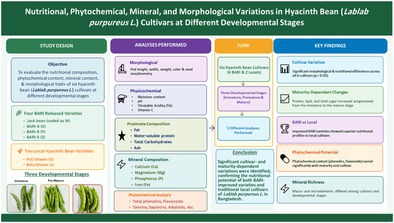

## 1 Introduction

Hyacinth bean (*Lablab purpureus L*.), commonly known as country bean, dolichos bean, or lablab bean, is an important leguminous vegetable crop belonging to the family Leguminosae. The crop is extensively cultivated in tropical and subtropical regions of Asia, Africa, and America, primarily for its tender green pods, which are consumed as vegetables (Shubha et al. 2024; Pandey et al. 2022). In Bangladesh, hyacinth bean, locally known as “deshi sheem,” is one of the most important indigenous winter vegetables, contributing substantially to household nutrition and national vegetable production (Rakibuzzaman et al. 2025; BBS 2023). The crop is widely grown in regions such as Cumilla, Noakhali, Sylhet, Dhaka, Tangail, Dinajpur, Jashore, and Chattogram, with a steadily increasing production trend in recent years (BBS 2023).

The green pods of hyacinth bean are nutritionally rich and affordable, making them an important dietary component, particularly in low‐ and middle‐income populations. Hyacinth bean pods contain appreciable amounts of carbohydrates, proteins, dietary fiber, vitamins (notably vitamin C and vitamin A), and essential minerals such as calcium, iron, phosphorus, and potassium (Deka and Sarkar 1990; Kilonzi 2017). In Bangladesh, where animal protein sources are often expensive and inaccessible to many, hyacinth bean pods serve as a valuable plant‐based protein source and are often referred to as “poor man's meat” (Kilonzi 2017; Ema et al. 2022).

Beyond their nutritional value, hyacinth bean pods contain various phytochemical constituents, including phenolics, flavonoids, tannins, saponins, and other bioactive compounds (Rakibuzzaman et al. 2025). These compounds are associated with antioxidant activity and potential health benefits, including protection against oxidative stress–related diseases (Rakibuzzaman et al. 2025). Hyacinth bean pods are also known for their excellent source of these micronutrients, which include calcium, potassium, phosphorus, iron, and many more. These minerals are essential for human growth, including bone development, energy metabolism, and oxygen transport (Fasoyiro et al. 2006). The presence of such bioactive compounds, together with essential minerals, highlights the potential of hyacinth bean pods as a nutrient‐rich vegetable crop. Both genetic background and environmental factors, such as soil fertility, agroclimatic conditions, and production methods, affect the accumulation of nutrients and phytochemicals in legumes. Numerous locally adapted landraces are grown alongside several improved cultivars created by the Bangladesh Agricultural Research Institute (BARI) in a variety of agro‐ecological zones throughout Bangladesh. Comparative data on compositional variation across locally accessible genotypes and improved cultivars, especially across developmental phases, are still scarce.

The nutritional composition of *L. purpureus* has been reported in several research, there is still a lack of comparative data on developmental‐stage‐dependent variation among readily available Bangladeshi landraces and improved BARI cultivars. Additionally, in Bangladeshi conditions, there is a lack of data integrating morphology, proximate composition, minerals, and phytochemicals across development stages.

Therefore, the current study sought to assess the variance in morphological attributes, proximate composition, phytochemical properties, and mineral content of certain hyacinth bean pods grown in Bangladesh based on genotype and developmental stage.

## 2 Methods and Materials

### 2.1 Sample Collection, Place, and Time

The four cultivars used in this study were Jackbean (coded as W), BARI‐6 (X), BARI‐8 (Y), and BARI‐5 (Z) that were collected from experimental fields where standard agricultural techniques were used. They had grown between October and December during a period of moderate weather. All experimental fields are supplied with appropriate fertiliser and soil management practices to achieve optimal crop production and maintain soil health. The samples obtained from the local market were representative of the local agricultural site or farmed area, where the landraces were grown using traditional farming methods in the Chattogram area.

**FIGURE 1:**
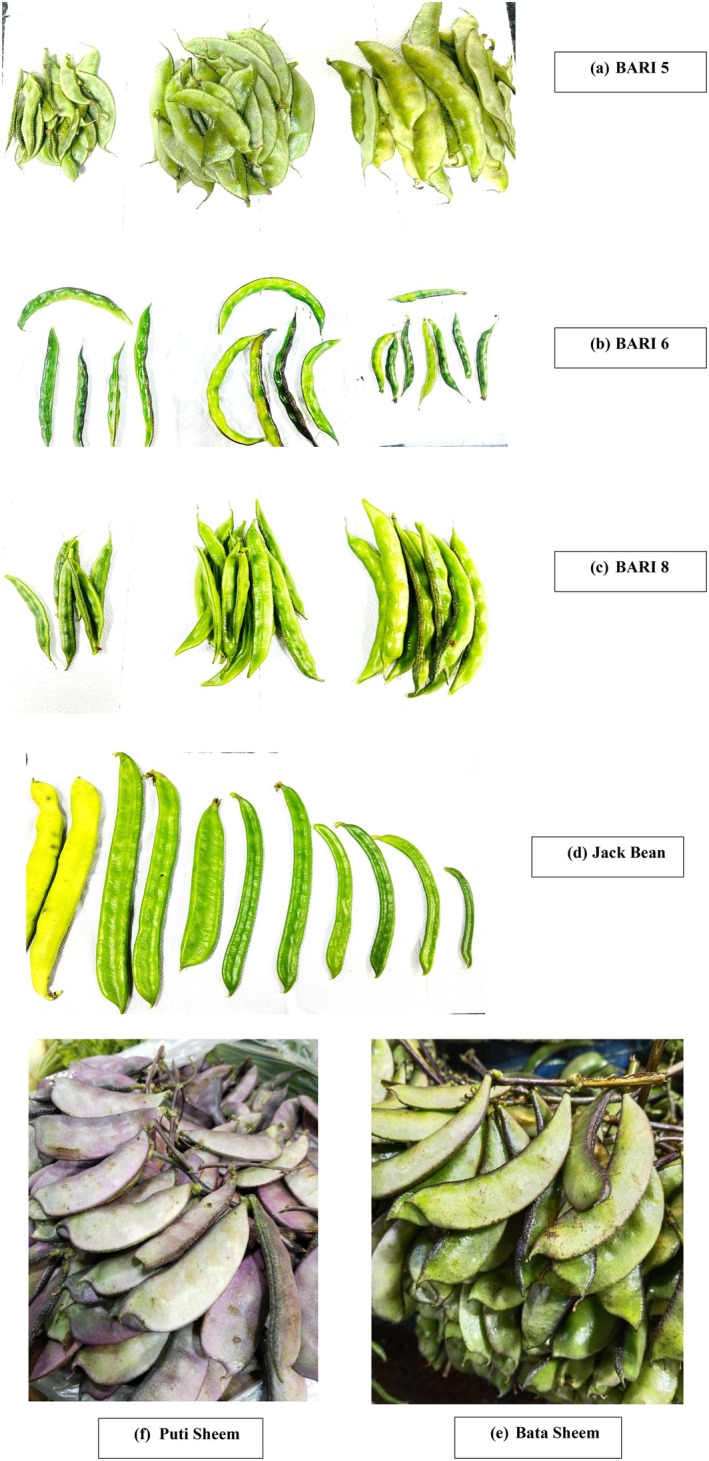
Representative images of six hyacinth bean ( *Lablab purpureus* L.) pod varieties used in this study. Four improved cultivars (Jack bean, BARI Seem‐5, BARI Seem‐6, and BARI Seem‐8) were collected from the Bangladesh Agricultural Research Institute (BARI), and two local landraces (Puti Sheem and Bata Sheem) were obtained from local markets in Chattogram. Pods from BARI cultivars were sampled at three developmental stages: immature, pre‐mature, and mature.

Local hyacinth bean varieties Puti Sheem (S) and Bata Sheem (L) were also found to be available in the local markets of Chattogram and are shown in Figure 1. All collected pods were washed thoroughly with distilled water and patted dry before analysis. For each cultivar and each stage of development, there are three biological replicates.

### 2.2 Sample Preparation

All the samples were properly cleaned with running tap water to remove dirt, then air‐dried. For additional preparation, the samples were split into two portions. One portion was chopped into small pieces, shade‐dried at ambient temperature, ground into fine powder using an electric grinder, and stored in sealed containers for further analysis. The other portion was cut into small pieces, mixed with 10–20 mL of distilled water, and the juice was extracted using a filter cloth for biochemical analysis.

### 2.3 Morphological Characterization of Pods

#### 2.3.1 Determination of Hyacinth Pod Weight

Fresh hyacinth bean pods were weighed individually using a calibrated electronic analytical balance (±0.001 g accuracy). The average weight was calculated from three replicates for each variety.

#### 2.3.2 Determination of Hyacinth Pod Length (cm) and Diameter

Using a digital Vernier caliper, the straight‐line distance between the style end and the pedicel (stalk end) was used to calculate the pod length (cm). At each pod's midpoint, the pod diameter (in centimeters) was measured. Three duplicates of each measurement were made, and the results were reported as mean ± SD.

### 2.4 Proximate and Physicochemical Analysis (Quantitative Analysis)

#### 2.4.1 Determination of Moisture Content

Moisture content of all the samples was determined by the Association of Official Analytical Chemists (AOAC 2000):

$$\text{Percent moisture content}\;\left(g/100\;g\;\text{sample}\right)=\text{Weight of moisture}\times 100/\text{Weight of sample taken}.$$

#### 2.4.2 Determination of Dry Matter Content

The percent dry matter content of the dry bean powder was calculated from the data obtained during moisture estimation using the following formula:

Percentage ofdrymatter=100–Percentage of moisture content.

#### 2.4.3 Determination of Total Soluble Solids (TSS) Content

TSS was determined by the digital refractometer (ATAGO Co., Tokyo, Japan) and expressed as degree Brix (°B). Approximately 3 g of the fresh sample was crushed to extract juice, and a single drop of the mixture was dropped onto the refractometer's prism for direct reading.

#### 2.4.4 Determination of pH Content

Two grams of hyacinth bean pods were homogenized well with 20 mL distilled water, filtered through Whatman's No. 1 filter paper, and centrifuged for 10 min at 4000 rpm. The pH of the supernatant was measured using a calibrated digital pH meter (Hanna Instruments, USA). Measurements were performed in triplicate.

#### 2.4.5 Determination of Water‐Soluble Protein Content (Lowry Method)

The protein content of all the hyacinth bean pod samples was determined by using bovine serum albumin (BSA) as a standard, using the Folin–Lowry method (Lowry et al. 1951). The absorbance was measured at 750 nm.

#### 2.4.6 Determination of Total Fat (Bligh and Dyer Method)

The total lipid content present in all hyacinth pod varieties was determined by using the Bligh and Dyer (1959) method.

#### 2.4.7 Determination of Total Sugar Content

The total sugar content of the samples was determined using the Anthrone method. The absorbance was measured at 620 nm Yemm and Willis (1954).

#### 2.4.8 Determination of Reducing Sugar Content

Reducing sugar content of bean juice was determined by the Dinitro‐salicylic acid method, and the absorbance was measured at 540 nm Miller (1959).

#### 2.4.9 Mineral Analysis

Mineral constituents (Ca, K, P, and Fe) were analyzed after wet acid digestion of the dried samples, following the AOAC (2016) methods. The digested samples were then analyzed using an atomic absorption spectrophotometer (Model AA‐7000, Shimadzu, Japan) (Isaac and Johnson 1975). The mineral content was quantified based on dry weight using standard formulas.

### 2.5 Quantitative Estimation of Phytochemicals

#### 2.5.1 Determination of Total Phenolic Content (TPC)

Total phenolic content was determined using the Folin–Ciocalteu reagent method and expressed as mg gallic acid equivalent (mg GAE)/100 g FW. Approximately 1 g of dried sample powder was extracted with 80% methanol for 24 h at room temperature before analysis (Harborne 1998).

#### 2.5.2 Determination of Total Flavonoid Content

Flavonoid content was estimated using the aluminum chloride (AlCl_3_) colorimetric method, and expressed as mg QE/100 g mg/100 g FW.

#### 2.5.3 Determination of Vitamin C

Vitamin C was determined using the 2,6‐dichlorophenolindophenol (DCPIP) titrimetric method.

### 2.6 Qualitative Analysis of Phytochemicals

#### 2.6.1 Test of Saponin

In a beaker, 30 mL of distilled water was mixed with 3 g of hyacinth pod powder. The mixture was heated in a water bath at 60°C to 70°C for 30 min. After filtering, 10 mL of the aqueous extract was transferred into a test tube, which was then violently shaken for 30 to 60 s. The presence of saponins is indicated by the formation of persistent foam.

#### 2.6.2 Test for Steroids

Two grams of hyacinth bean pods powder was dissolved in 10 mL of methanol. The solvent was vortexed for 30 min and centrifuged at 5000 RPM for 10 min. Slowly, 1 mL of concentrated H_2_SO_4_ was added, and finally, the interface was examined to see if it formed a bluish‐green ring.

#### 2.6.3 Test for Coumarin

The alkaline reagent test was used to qualitatively detect whether coumarins were present. Five grams of hyacinth pods powder were dissolved in 20 mL of 95% ethanol, it was shaken well to mix properly, and then left to stand for 30 min, and heated in a water‐bath at 50°C to 60°C for 15 min.2 to 3 drops of dilute HCl were added to the mixture, and the color changes indicate the presence of coumarins.

#### 2.6.4 Test for Terpenoids

Two grams of dried pod powder is dissolved in 10 mL of chloroform. The mixture was shaken well and left to stand for 30 min. Then 2 mL chloroform extract was taken, and 2 mL of concentrated H_2_SO_4_ was added. The presence of a red‐brown interface indicates the presence of terpenoids.

### 2.7 Statistical Analysis

Every experiment was conducted in triplicate, and the mean ± standard deviation (SD) was used to express the results. Two‐way analysis of variance (ANOVA) was used in the statistical analysis to assess how genotype and developmental stage affected the measured parameters. At *p* < 0.05, differences were considered statistically significant.

## 3 Results and Discussion

Table S1 presents a summary of the two‐way ANOVA findings illustrating the impact of developmental stage and genotype on particular parameters.

### 3.1 Morphological Characteristics of Pods

Significant differences (*p* < 0.05) were found for pod length, width, and weight between cultivars and development stages (Table 1). The length of the pods varied from 4.0 cm (immature BARI‐5) to 28.30 cm (mature Jackbean) cultivars, with a weight variation from 0.23 to 65.23 g. Mature stage pods showed considerable differences for all characteristics when compared to premature and immature stages, suggesting progressive increases in size and mass with different maturity stages and also reflecting enhanced cell expansion, dry matter deposition, and seed filling.

**TABLE 1:** Morphological characteristics of hyacinth bean ( *Lablab purpureus* L.) pods at different developmental stages.

Variety	Pod stage	Length (cm)	Width (cm)	Weight (g)
BARI‐5 (Z)	Mature	12.0 ± 0.02^a^	2.9 ± 0.05^a^	9.1 ± 0.01^a^
	Pre‐mature	8.0 ± 0.01^b^	1.8 ± 0.02^b^	2.1 ± 0.01^b^
	Immature	4.0 ± 0.06^c^	0.5 ± 0.01^c^	0.2 ± 0.01^c^
BARI‐8 (Y)	Mature	15.0 ± 0.02^a^	1.7 ± 0.02^a^	12.8 ± 0.01^a^
	Pre‐mature	12.0 ± 0.02^b^	1.3 ± 0.01^b^	4.2 ± 0.0^b^
	Immature	6.8 ± 0.02^c^	1.0 ± 0.10^c^	1.1 ± 0.02^c^
BARI‐6 (X)	Mature	16.5 ± 0.02^b^	1.8 ± 0.02^a^	13.7 ± 0.06^a^
	Pre‐mature	13.5 ± 0.058^a^	1.4 ± 0.01^b^	8.2 ± 0.06^b^
	Immature	10.0 ± 0.02^c^	0.9 ± 0.02^c^	2.4 ± 0.02^c^
Jack bean (W)	Mature	28.3 ± 0.18^a^	3.6 ± 0.01^a^	65.2 ± 1.37^a^
	Pre‐mature	26.7 ± 0.39^b^	2.8 ± 0.01^b^	47.5 ± 0.11^b^
	Immature	19.2 ± 0.10^c^	1.7 ± 0.01^c^	15.4 ± 0.14^c^
Bata Sheem (L)	Mature	9.3 ± 0.011^a^	2.7 ± 0.01^a^	9.6 ± 0.09^a^
Puti Sheem (S)	Mature	11.20 ± 0.05^a^	2.81 ± 0.01^a^	14.67 ± 0.40^a^

*Note:* Values are expressed as mean ± SD (*n* = 3). Different superscript letters within the same column indicate significant differences at *p* < 0.05 according to Tukey's HSD test.

This form of developmental increase in pod size and mass has been noted for other leguminous vegetables, with biomass development correlated to filling of seeds (Maass et al. 2010). Similar observations have been commonly noted for hyacinth bean, with a reference to the increase in size and mass attributed to factors like genetics and developmental stage (Maass et al. 2010). The large pod size in the Jack bean implies a superior yield potential, whereas the smaller pods at immature stages are desirable for tender vegetable consumption due to low fiber content.

### 3.2 Proximate Composition of Different Varieties of Hyacinth Bean Pods

Table 2 represents the results of the physiochemical and proximate composition of all hyacinth bean pods at different developmental stages. Significant genotype‐dependent variation in moisture content was observed (*p* < 0.05), whereas developmental stage showed no significant effect under the two‐way ANOVA model. This is because moisture is a critical factor affecting post‐harvest stability and nutritional density (Rickman et al. 2007).

**TABLE 2:** Physicochemical and proximate composition of hyacinth bean ( *Lablab purpureus* L.) pods from different genotypes and developmental stages (fresh weight basis).

Variety	Pod stage	Moisture (%)	Dry matter (%)	TSS (°Brix)	pH	Water‐soluble protein content (g/100 g FW)	Total fat (g/100 g FW)	Total sugar (g/100 g FW)	Reducing sugar (g/100 g FW)
Jack bean (W)	Mature	23.6 ± 0.10^b^	76.4 ± 0.10^b^	9.4 ± 0.01^a^	6.6 ± 0.10^c^	0.016 ± 0.003^b^	0.64 ± 0.02^a^	1.73 ± 0.25^b^	1.33 ± 0.40^a^
	Premature	23.8 ± 0.05^a^	76.2 ± 0.05^c^	5.4 ± 0.02^b^	7 ± 0.10^b^	0.034 ± 0.001^a^	0.58 ± 0.02^b^	2.14 ± 0.56^a^	0.76 ± 0.25^c^
	Immature	16.3 ± 0.10^c^	83.7 ± 0.10^a^	4.3 ± 0.01^c^	7.5 ± 0.01^a^	0.01 ± 0.001^c^	0.40 ± 0.03^c^	1.22 ± 0.31^c^	1.14 ± 0.46^b^
BARI‐5 (Z)	Mature	25.3 ± 0.59^a^	74.7 ± 0.59^c^	1.6 ± 0.02^a^	6.00 ± 0.10^c^	0.018 ± 0.001^c^	0.123 ± 0.01^c^	3.55 ± 0.60^a^	2.33 ± 0.36^a^
	Premature	21.5 ± 0.42^b^	78.5 ± 0.42^b^	1.2 ± 0.01^b^	6.1 ± 0.11^a^	0.03 ± 0.004^a^	0.18 ± 0.02^b^	2.53 ± 0.35^b^	1.18 ± 0.72^b^
	Immature	21.2 ± 0.52^c^	78.8 ± 0.52^a^	0.9 ± 0.02^c^	6.0 ± 0.06^b^	0.02 ± 0.002^b^	0.19 ± 0.01^a^	1.83 ± 0.32^c^	0.64 ± 0.41^c^
BARI‐6 (X)	Mature	25.2 ± 0.06^c^	74.9 ± 0.06^a^	1.1 ± 0.02^a^	6.1 ± 0.06^b^	0.012 ± 0.002^c^	0.25 ± 0.02^a^	3.06 ± 0.70^a^	1.75 ± 0.46^a^
	Premature	26.7 ± 0.02^a^	73.3 ± 0.02^c^	0.9 ± 0.01^b^	6.0 ± 0.01^c^	0.055 ± 0.002^a^	0.01 ± 0.01^c^	2.42 ± 0.30^b^	1.17 ± 0.36^b^
	Immature	25.6 ± 0.61^b^	73.7 ± 0.61^b^	0.7 ± 0.01^c^	7.0 ± 0.01^a^	0.022 ± 0.002^b^	0.17 ± 0.01^b^	1.03 ± 0.58^c^	0.89 ± 0.20^c^
BARI‐8 (Y)	Mature	10.5 ± 0.04^c^	84.9 ± 0.04^c^	0.8 ± 0.01^b^	7.01 ± 0.06^b^	0.024 ± 0.0015^c^	0.29 ± 0.02^b^	1.81 ± 0.21^b^	1.71 ± 0.36^a^
	Premature	10.5 ± 0.21^b^	86.7 ± 0.21^a^	1.2 ± 0.02^a^	7 ± 0.01^a^	0.03 ± 0.002^a^	0.35 ± 0.01^a^	2.52 ± 0.26^a^	1.34 ± 0.44^b^
	Immature	15.8 ± 0.10^a^	89.5 ± 0.10^b^	1.0 ± 0.02^c^	6.7 ± 0.01^c^	0.03 ± 0.002^b^	0.27 ± 0.02^c^	1.23 ± 0.34^c^	0.94 ± 0.45^c^
Puti sheem (S)	Mature	16.0 ± 0.16^a^	83.8 ± 0.16^b^	1.6 ± 0.01^a^	6.0 ± 0.02^a^	0.03 ± 0.002^b^	0.03 ± 0.01^b^	2.33 ± 0.32^a^	1.34 ± 0.57^b^
	Premature	13.6 ± 0.10^b^	86.4 ± 0.10^a^	1.2 ± 0.01^b^	6.0 ± 0.01^b^	0.014 ± 0.001^a^	0.06 ± 0.32^a^	1.51 ± 0.25^b^	1.47 ± 0.24^a^
Bata Sheem (L)	Mature	22.3 ± 0.10^a^	77.7 ± 0.10^a^	2.4 ± 0.01^b^	5.4 ± 0.01^a^	0.014 ± 0.001^b^	0.014 ± 0.001^b^	2.26 ± 0.95^a^	2.17 ± 0.31^a^
	Premature	23.0 ± 0.06^b^	76.9 ± 0.06^b^	5.4 ± 0.01^a^	5.4 ± 0.01^b^	0.034 ± 0.002^a^	0.034 ± 0.002^a^	1.82 ± 0.26^b^	1.56 ± 0.64^b^

*Note:* Values are presented as mean ± SD (*n* = 3). Different superscript letters within a column indicate significant differences at *p* < 0.05 according to Tukey's HSD test.

Abbreviation: FW, fresh weight.

Significant variations in moisture content were more closely linked to genotype than developmental stage. The moisture content of BARI‐8 was lower than that of BARI‐6 and Jack bean, suggesting cultivar‐dependent water retention traits (Boye et al. 2010). The stage effect was not statistically significant under the two‐way ANOVA model, despite the fact that numerical variance was seen between developmental stages.

According to the two‐way ANOVA model, pod pH did not substantially vary between genotypes or developmental phases (*p* > 0.05), suggesting that acidity was generally consistent between cultivars. Vegetable legumes have shown similar pH stability throughout pod growth, which may be linked to balanced metabolism of organic acids and carbohydrates (Lee and Kader 2000; Ahmed et al. 2011).

Protein content varied little between developmental stages and genotypes. The very constant patterns of nitrogen buildup during pod formation may be linked to the little variance seen. Similar results have been shown for leguminous plants, where the protein concentration is relatively constant throughout the maturity stages (Boye et al. 2010).

Lipid content exhibited significant variation (*p* < 0.05), but remained low, consistent with fresh vegetable legumes where lipids contribute minimally to caloric value (Siddhuraju and Becker 2003).

Total soluble solids (TSS) did not differ significantly among genotypes or maturity stages (*p* > 0.05), indicating relative stability in soluble solids during pod development.

Both total sugar (*p* = 0.33) and reducing sugar (*p* = 0.23) did not differ significantly (*p* > 0.05) between genotypes or developmental stages (Table 2). These results show that during pod development, soluble carbohydrate accumulation is comparatively constant across cultivars. Compared to the more variable mineral and phytochemical components, sugar partitioning seemed to be preserved despite slight variations. Other leguminous vegetables have been shown to have comparable stability in their sugar concentration (Keskin et al. 2021).

Overall, two‐way ANOVA indicated that genotype had a greater influence on most compositional parameters than developmental stage.

### 3.3 Mineral Composition

Significant genotype × developmental stage interactions were observed for several minerals, indicating differential nutrient accumulation patterns among cultivars during pod maturation (Table 3).

**TABLE 3:** Mineral composition of hyacinth bean ( *Lablab purpureus* L.) cultivars at different maturity stages (mg/kg DW).

Variety	Pod stage	Ca (mg/kg DW)	Fe (mg/kg DW)	K (mg/kg DW)	P (mg/kg DW)
Jack bean (W)	Mature	721.33 ± 1.53^b^	21.2 ± 1.6^b^	1564.7 ± 2.1^c^	200.3 ± 2.5^c^
	Premature	660.00 ± 1.1^c^	22.6 ± 1.1^a^	1955.3 ± 1.5^b^	399.7 ± 1.5^b^
	Immature	790.00 ± 1.0^a^	19.3 ± 0.7^c^	2346.0 ± 2.0^a^	401.7 ± 2.1^a^
BARI‐6 (X)	Mature	1440.00 ± 1.0^a^	17.4 ± 0.7^c^	2344.7 ± 3.2^a^	299.3 ± 2.1^c^
	Premature	520.33 ± 1.5^c^	20.7 ± 0.9^b^	1951.7 ± 3.1^b^	402.7 ± 2.5^a^
	Immature	1130.67 ± 2.1^b^	21.8 ± 0.9^a^	785.7 ± 3.5^c^	400.0 ± 1.0^b^
BARI‐5 (Z)	Mature	1030.00 ± 2.0^b^	19.3 ± 0.8^a^	782.0 ± 3.0^c^	100.3 ± 1.5^c^
	Premature	1039.67 ± 1.5^a^	17.7 ± 0.9^c^	1175.3 ± 2.5^b^	299.7 ± 1.5^b^
	Immature	450.00 ± 1.0^c^	18.4 ± 0.7^b^	1564.0 ± 2.0^a^	501.3 ± 3.2^a^
BARI‐8 (Y)	Mature	901.00 ± 1.0^a^	18.4 ± 0.6^c^	1954.0 ± 2.7^a^	200.7 ± 2.1^c^
	Premature	551.33 ± 1.5^c^	21.4 ± 0.9^b^	1171.7 ± 1.5^c^	400.7 ± 3.1^b^
	Immature	740.33 ± 1.5^b^	21.7 ± 0.8^a^	1565.3 ± 3.2^b^	501.00 ± 3.6^a^
Bata Sheem (S)	Mature	960.33 ± 1.5^b^	17.3 ± 0.7^b^	780.3 ± 1.5^b^	299.7 ± 2.5^b^
	Premature	1430.67 ± 2.1^a^	28.6 ± 0.5^a^	1174.3 ± 3.2^a^	499.7 ± 1.5^a^
Puti Sheem (L)	Mature	1280.00 ± 1.0^a^	18.7 ± 0.4^b^	783.7 ± 2.1^b^	302.00 ± 2.7^b^
	Premature	1230.00 ± 1.1^b^	25.6 ± 0.4^a^	1175.3 ± 2.1^a^	801.00 ± 1^a^

*Note:* Values are presented as mean ± SD (*n* = 3). Different superscript letters within a column indicate significant differences at *p* < 0.05 according to Tukey's HSD test.

Abbreviation: DW, dry weight.

Potassium was the major mineral component (780.3–2346.0 mg/kg DW), with higher concentrations observed at early stages of development, probably due to its role in osmotic regulation and fast cell growth (Mubarak 2005).

Calcium contents ranged from 450.0 to 1440.0 mg/kg DW and increased with maturity, probably due to its role in cell wall stabilization (Fasoyiro et al. 2006). Phosphorus (100.3–802.0 mg/kg DW) and iron (17.3–28.6 mg/kg DW) contents were within the ranges reported for legume pods (Iqbal et al. 2006).

These variations may be due to differences in nutrient uptake efficiency among genotypes, as well as to environmental factors such as soil mineral availability and fertilization practices. These results may be due to genotype‐environment interactions, especially considering the presence of BARI cultivars grown at research stations as well as local varieties grown under varying conditions.

### 3.4 Bioactive Compounds

As shown in Table 4, genotype‐dependent variation in phenolic accumulation was indicated by the considerable differences in total phenolic content between cultivars. Increased secondary metabolite biosynthesis linked to growth‐phase defensive mechanisms may be the cause of higher phenolic concentrations in early developmental phases (Xu and Chang 2007).

**TABLE 4:** Genotype‐ and maturity‐dependent variation in phenolic content, flavonoid content, and vitamin C of hyacinth bean pods.

Variety	Pod stage	Phenolics (mg/100 g FW)	Flavonoid (mg/100 g FW)	Vitamin C (mg/100 g FW)
Jack bean (W)	Mature	22.16 ± 0.63^b^	698 ± 3.21^a^	5.57 ± 0.10^a^
	Premature	26.67 ± 0.35^a^	613 ± 1.36^b^	5.35 ± 0.08^b^
	Immature	28.46 ± 0.27^a^	433.6 ± 2.52^c^	5.29 ± 0.02^c^
BARI‐5 (Z)	Mature	20.91 ± 0.11^c^	407.7 ± 2.08^a^	6.15 ± 0.02^c^
	Premature	24.35 ± 0.23^b^	369.7 ± 1.53^b^	6.32 ± 0.03^b^
	Immature	26.00 ± 0.59^a^	315.6 ± 2.55^c^	6.44 ± 0.10^a^
BARI‐6 (X)	Mature	17.95 ± 0.28^a^	663.03 ± 4.05^b^	5.31 ± 0.07^c^
	Premature	16.86 ± 0.23^b^	347.3 ± 2.08^c^	5.35 ± 0.07^b^
	Immature	17.81 ± 0.13^c^	885.3 ± 1.53^a^	5.39 ± 0.02^a^
BARI‐8 (Y)	Mature	30.13 ± 0.28^a^	450.3 ± 1.08^c^	6.46 ± 0.08^a^
	Premature	25.34 ± 0.49^c^	580.3 ± 0.46^b^	6.28 ± 0.05^b^
	Immature	28.64 ± 0.25^b^	707 ± 0.32^a^	6.16 ± 0.05^c^
Puti Sheem (S)	Mature	27.13 ± 0.68^a^	581.7 ± 2.06^b^	6.34 ± 0.06^b^
	Premature	26.46 ± 0.34^b^	588.1 ± 1.95^a^	6.39 ± 0.02^a^
Bata Sheem (L)	Mature	20.9 ± 0.15^a^	723.0 ± 8.0^b^	5.62 ± 0.07^a^
	Premature	19.28 ± 0.20^b^	785 ± 1.73^a^	5.29 ± 0.02^b^

*Note:* Values are presented as mean ± SD (*n* = 3). Different superscript letters within a column indicate significant differences at *p* < 0.05 according to Tukey's HSD test.

Abbreviation: FW, fresh weight.

Strong genotype‐dependent variation in secondary metabolite accumulation was indicated by the considerable differences in flavonoid concentration between genotypes. Flavonoids play a significant role in antioxidant activity and are usually abundant throughout early growth (Heim et al. 2002) (Singleton et al. 1999).

Significant differences in vitamin C content were found between genotypes, indicating that genetic background rather than developmental stage is the main factor influencing ascorbic acid accumulation.

Both genetic regulation of secondary metabolism and environmental modification, such as exposure to stress, soil composition, and culture methods, can lead to variations in phytochemical accumulation.

Genotype was the primary factor impacting the majority of biochemical markers, according to two‐way ANOVA, while developmental stage had no statistical significance in the present procedure. These results imply that compositional variability is more significantly influenced by genetic background than by pod maturity alone.

### 3.5 Qualitative Phytochemical Screening

The qualitative phytochemical screening confirmed the presence of major bioactive compounds, which include steroids, tannins, saponins, and coumarins across all the varieties, as depicted in Table 5. Differential secondary metabolite production influenced by genotype and maturity stage is suggested by variations in detection intensity. For plant defense, secondary metabolite accumulation is developmentally controlled and frequently increased during early growth stages (Treutter 2005). The consistent detection of these phytochemicals indicates the nutraceutical value of these pods.

**TABLE 5:** Qualitative phytochemical screening of different hyacinth bean pod varieties.

Variety	Saponin	Steroids	Coumarins	Tannins
Jack bean (W)	+	+	+	+
BARI‐5 (Z)	+	+	+	+
BARI‐6 (X)	+	+	+	+
BARI‐8 (Y)	+	+	+	+
Puti Sheem (S)	+	+	+	+
Bata Sheem (L)	+	+	+	+

*Note:* (+) indicates presence; (−) indicates absence.

## 4 Limitations

Without sophisticated antioxidant assays or compound‐specific profiling, the investigation was restricted to specific minerals and broad phytochemical estimations. Further research using chromatographic characterization, antioxidant activity tests, and multi‐location trials might shed more light on the nutritional quality of different genotypes.

## 5 Conclusions

The morphological, nutritional, mineral, and phytochemicals of the hyacinth bean pods varied considerably in the various genotypes and developmental stages of several Bangladeshi cultivars. The mature pods had a dry matter content and total mineral content that were greater than those found in the immature or premature pods; however, at the same time, the immature and premature pods contained more phenolics, flavonoids, and vitamin C than the mature pods. Potassium was found to be the most abundant mineral in the pods of all the hyacinth bean cultivars; however, all other minerals were calcium, phosphorus, and iron.

Two‐way ANOVA analysis showed that the genotype had a greater effect on the majority of variables than did the developmental stage of the pod. Phytochemical screening showed there were several bioactive compounds present in all of the genotypes of the hyacinth bean.

The findings from this research provide evidence of the importance of selecting appropriate cultivars and harvest stages to optimize nutritional quality and encourage the use of hyacinth bean as a high‐nutrient vegetable crop in Bangladesh. More advanced antioxidant assay studies, additional compound analysis, and multi‐site trials should be conducted to further validate and expand this research.

## Author Contributions

**Rimon Barua:** resources, investigation. **Ahmed Saiyadeen Nusaha:** software, formal analysis. **Gazi Wafa Akbar:** writing – original draft, supervision. **Tania Ismail:** methodology, investigation, project administration. **Mubtasim Adnan:** investigation, formal analysis. **Noshin Nower Bristy:** investigation, methodology, project administration. **Jobed Nahar:** investigation. **Afroze Anwar:** investigation. **Nurul Absar:** conceptualization. **Sabrina Mansur:** visualization, investigation.

## Funding

This research was supported by a research grant from the University Research Cell (URC), University of Science and Technology Chittagong (USTC). The funding body had no role in study design, data collection, analysis, interpretation, or manuscript preparation.

## Disclosure

All authors have read and approved the final version of the manuscript. Gazi Wafa Akbar had full access to all data in this study and takes full responsibility for the data's integrity and the accuracy of the analysis.

## Ethics Statement

The authors have nothing to report.

## Conflicts of Interest

The authors declare no conflicts of interest.

## Supporting information


**Table S1:** Summary of two‐way ANOVA results showing the effects of genotype and developmental stage on selected biochemical parameters of hyacinth bean pods.

## Data Availability

The authors confirm that the data supporting the findings of this study are available within the article and its Supporting Information.
