# Supplementary material for: Nutritional, Phytochemical, Mineral, and Morphological Variations in Hyacinth Bean (Lablab purpureus L.) Cultivars at Different Developmental Stages
Source: Food Sci Nutr. 2026 Jul 6;14(7):e72086. doi: 10.1002/fsn3.72086 (PMC13335611; doi:10.1002/fsn3.72086)
Supplement: Supplementary file 1 — Table S1: Summary of two‐way ANOVA results showing the effects of genotype and developmental stage on selected biochemical parameters of hyacinth bean pods. [file FSN3-14-e72086-s001.docx]

| Parameter | Genotype Effect | Developmental Stage Effect |
| --- | --- | --- |
| Moisture Content | Significant (p < 0.01) | NS |
| Protein Content | NS | Significant (p < 0.05) |
| Vitamin C | Significant (p < 0.001) | NS |
| Lipid Content | Significant (p < 0.01) | NS |
| Total Flavonoid Content | Significant (p < 0.001) | NS |
| pH | NS | NS |

**Table S1: Summary of two-way ANOVA results showing the effects of genotype and developmental stage on selected biochemical parameters of hyacinth bean pods.**
